# Exposure to the herbicide glyphosate leads to inappropriate threat responses and alters gut microbial composition

**DOI:** 10.3389/ftox.2025.1704231

**Published:** 2025-11-05

**Authors:** Mauricio Cáceres-Chacón, Osmarie Martínez-Guzmán, Héctor A. Haddock-Martínez, Alexdiel Figueroa-Pérez, Sian Rodríguez-Rosado, Jaleniz Suárez-Pérez, Raúl Y. Ramos-Sánchez, Filipa Godoy-Vitorino, Demetrio Sierra-Mercado

**Affiliations:** ^1^ Department of Anatomy and Neurobiology, University of Puerto Rico School of Medicine, San Juan, PR, United States; ^2^ Department of Microbiology and Medical Zoology, University of Puerto Rico School of Medicine, San Juan, PR, United States

**Keywords:** mental health disorders, classical conditioning, bed nucleus of stria terminalis, learning and memory, rodent, *Lactobacillus*

## Abstract

**Background:**

Exposure to the herbicide glyphosate may contribute to anxiety-related disorders. The mechanisms by which this occurs may involve effects on brain regions or alterations of gut microbiota implicated in emotions. Pre-clinical studies use unusually high doses to which humans may not normally be exposed. The effects of glyphosate on anxiety at doses considered safe are largely unexplored.

**Methods:**

Adult male rats were administered glyphosate at a dose considered safe for 16 weeks. After 4 and 10 weeks, anxiety-like behaviors were tested in the open field and elevated plus maze, respectively. After 14 weeks, rats interacted with a novel neutral object, followed by a naïve rat of the same age and sex as a conspecific. Lastly, after 16 weeks, rats underwent fear conditioning, and freezing was quantified. Upon completion of the experiments, cellular activity was assessed using immunohistochemistry in brain regions implicated in anxiety and fear. Fecal pellets were collected to extract DNA and perform 16S rRNA community analyses.

**Results:**

Glyphosate increases anxiety in the elevated plus maze after 10 weeks. Moreover, glyphosate decreases interaction to a novel object, but not to the conspecific, after 14 weeks. Furthermore, freezing increases to a novel neutral tone, but not a conditioned tone, after 16 weeks. Behavioral changes correspond to increases in cellular activity in the bed nucleus of the stria terminalis. Furthermore, we observed that glyphosate induces changes in the gut microbiota leading to a decrease in *Lactobacillus* species.

**Conclusion:**

Glyphosate increases threat interpretation and alters cellular activity in brain regions implicated in promoting anxiety. Also, glyphosate induces gut dysbiosis and reduces the abundance of *Lactobacillus*, bacteria that play a role in the production of serotonin, which may further exacerbate the anxiogenic effect of glyphosate.

## Introduction

Anxiety and fear disorders can develop across the lifespan and are characterized by hypervigilance in situations that do not warrant it (Ohi et al., 2025). Exposure to environmental contaminants may contribute to the development of hypervigilance. A contaminant that has received attention due to its effect on health is the herbicide glyphosate (Gandhi et al., 2021; Costas-Ferreira et al., 2022; Ojelade et al., 2022).

Glyphosate revolutionized commercial herbicides (i.e., Roundup®) because it was thought to be innocuous to humans, as it targets an enzyme not found in mammals. However, clinical reports demonstrate that glyphosate-based herbicides can cross the blood brain barrier and affect the brain (Eriguchi et al., 2019; Planche et al., 2019). Furthermore, Seneff and colleagues (2015) suggested a correlation between the use of glyphosate-based herbicides and the diagnosis of anxiety and fear-related disorders. Similarly, preclinical work in rodents demonstrates that glyphosate increases anxiety-like behaviors (Ait Bali et al., 2017; 2018; 2022). Remarkably, these studies utilized unusually high doses of up to 500 mg/kg/day to which humans are unlikely to be exposed (Ait Bali et al., 2017; 2018; 2022). In fact, the Environmental Protection Agency (EPA) established a chronic reference dose of 2.0 mg/kg/day for prolonged periods as safe for humans (EPA, 2017). The effects of glyphosate at doses considered safe on anxiety-like and fear-related behaviors are largely unexplored.

Glyphosate has been shown to increase cellular activity in brain regions that promote the expression of anxiety-like behaviors (Ait Bali et al., 2022). The basolateral amygdala (BLA) creates associations between neutral stimuli and aversive outcomes, which initiates threat responses (LeDoux, 2014). Activity in bidirectional projections between the BLA and the medial prefrontal cortex (mPFC) enhances the expression of anxiety and fear (Kredlow et al., 2022). The infralimbic (IL) subregion of the mPFC increases the expression of anxiety-like behaviors (Bi et al., 2013) likely via connections with the bed nucleus of the stria terminalis (BNST), whereas the prelimbic (PL) subregion drives the expression of conditioned fear (Sierra-Mercado et al., 2011). The serotonergic system regulates anxiety-like and fear-related behaviors by modulating activity in PFC and BNST (Albert et al., 2014; Almada et al., 2015; García-García et al., 2018; Yamashita et al., 2019). Interestingly, alterations in gut microbiota composition affect anxiety and fear (Hoban et al., 2018; Chu et al., 2019; Geary et al., 2021). Specifically, bacterial genera such as *Lactobacillus* affect emotional behaviors by modulating levels of serotonin (Bravo et al., 2011; Cowan et al., 2019). Indeed, glyphosate has been shown to impact gut microbiome (Lozano et al., 2018; Mao et al., 2018; Tang et al., 2020b), and this may be a mechanism through which it affects anxiety-like and fear-related behaviors (Ait Bali et al., 2018; Lozano et al., 2018; Dechartres et al., 2019).

We hypothesized that prolonged exposure to glyphosate at a dose considered safe (EPA, 2017) would increase anxiety-like and fear-related behaviors, as well as increase cellular activity in respective regions of the brain, given the overlapping mechanisms of anxiety and fear. We also hypothesized that glyphosate would reduce abundance of gut bacteria implicated in the production of serotonin. The results help in understanding how environmental contaminants could influence mental health disorders.

## Materials and methods

### Animals

A total of 29 adult male Sprague-Dawley rats (Envigo+, IN, United States, ∼325–350 g upon arrival) were individually housed on a 12:12 light/dark cycle. Of the 29 rats, 25 rats were used for all behavioral tests, whereas four rats were used as conspecifics in a Social Exploration Test. These four conspecific rats were not used in any other experiment in the study. Behavioral experiments were run between 9a.m. and 1p.m. Rats were randomly assigned to their study group. Rats were handled for 7 days to acclimate them to the experimenter. All procedures were approved by the Institutional Animal Care and Use Committee of the University of Puerto Rico Medical Sciences Campus, which is accredited by the Association for Assessment and Accreditation of Laboratory Animal Care International (AAALAC). Thus, environmental parameters, such as light intensity, background noise, temperature, and humidity are constantly monitored and maintained to the highest standards for animal welfare throughout the study. Experiments were performed by investigators blinded to the groups.

### Bar-press training

Rats were food-restricted to 18g/day. They were then trained to press a lever for sucrose pellets in an operant chamber on a variable interval schedule of reinforcement, receiving one pellet every 60 s regardless of the number of presses (VI-60). Rats achieved a criterion of ∼10 presses per minute. Food restriction was discontinued, and food was given *ad libitum*. Lever pressing maintains the rat active so that freezing can be measured with confidence during fear conditioning (Sierra-Mercado et al., 2006; 2011).

### Glyphosate solution and exposure protocol

Glyphosate solution was prepared weekly to ensure stability, even though the half-life of glyphosate in water is greater than 60 days (Bonnet et al., 2007). Rats were given access *ad libitum* to either glyphosate-containing water or filtered water for controls for 16 weeks. Baseline water consumption was measured for 3 weeks to determine average water intake. Average concentrations of glyphosate (PESTANAL®, Sigma-Aldrich, MO, United States) per rat were calculated to provide an average daily dose of 2.0 mg/kg. Body weight and water intake were measured at the end of each week, and if needed, the concentration of glyphosate was adjusted accordingly to ensure an average dose of 2.0 mg/kg/day was obtained.

### Anxiety-like behaviors

**FIGURE 1:**
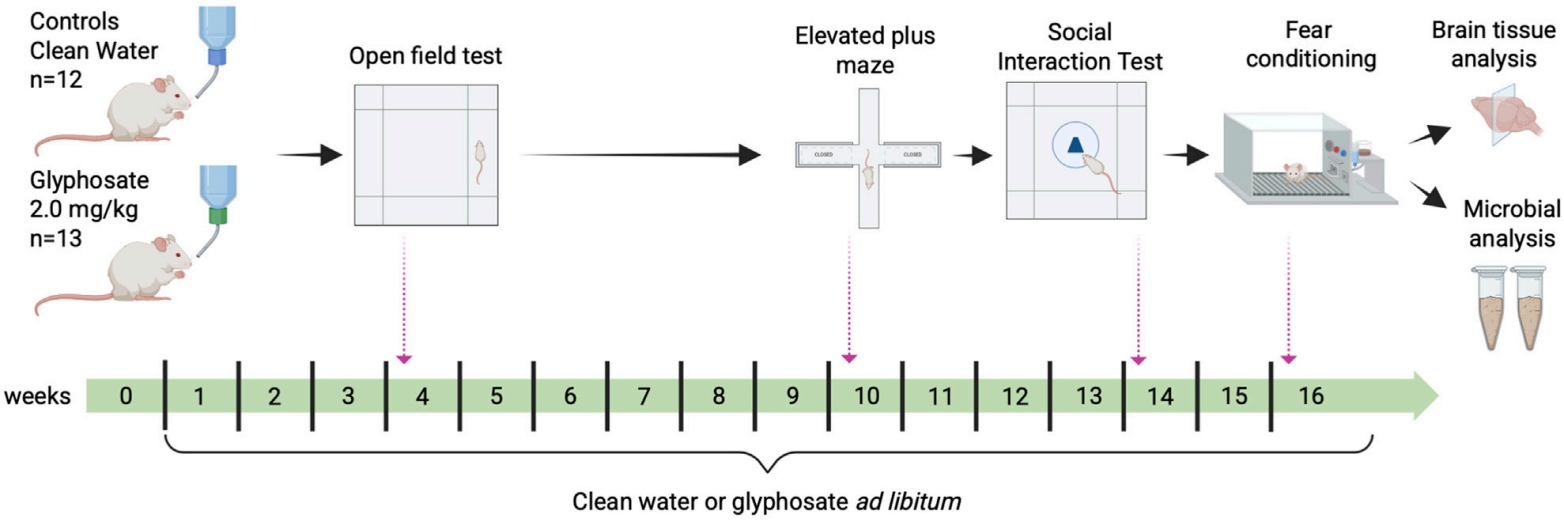
Experimental design and timeline. Experimental rats were exposed to glyphosate 2.0 mg/kg/day for 16 weeks, whereas controls were given filtered water. Behavioral tests were assessed at specific time points throughout the exposure period: Open Field Test (week 4), Elevated Plus Maze (week 10), Social Exploration Test (Week 14) and Fear Conditioning (week 16). Fecal pellets were collected on week 16 of exposure to assess for changes in gut microbiome. All rats were trained to press a lever for sucrose pellets necessary for the fear conditioning experiment prior to any manipulations.

**FIGURE 2:**
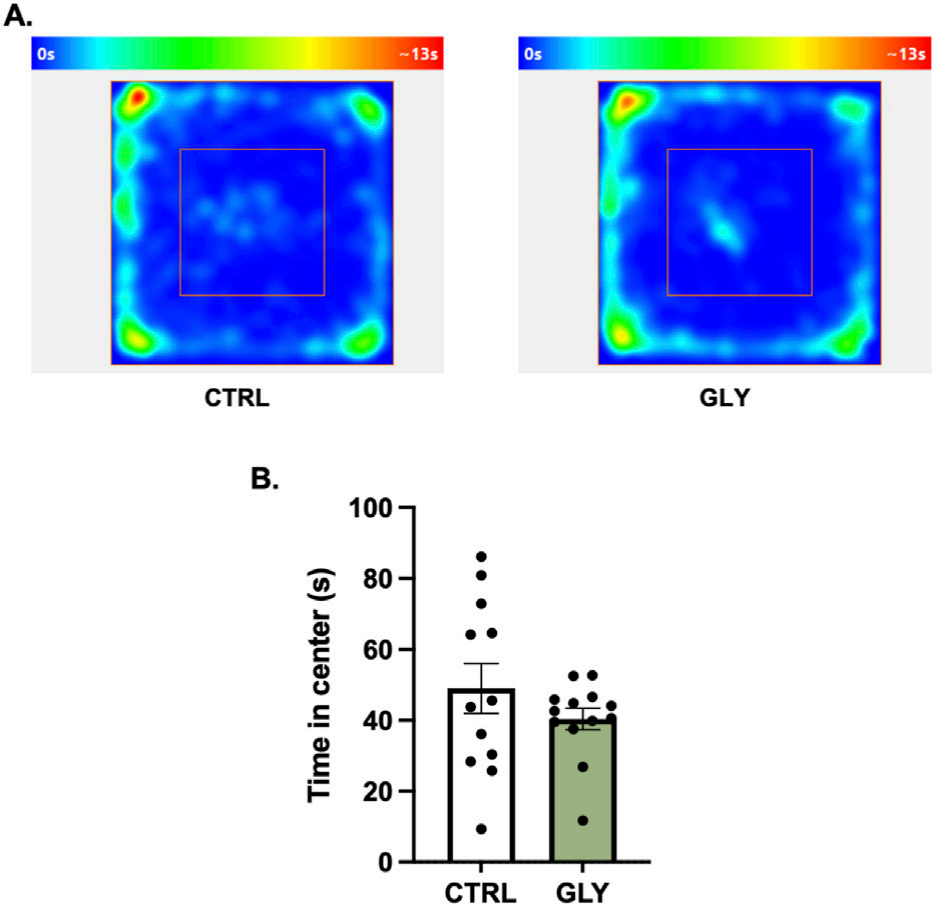
**(A)** Glyphosate after 4 weeks does not affect anxiety-like behaviors in an open field test. **(A)** Average heat maps showing position of the animals. **(B)** Time spent in the center (CTRL: n = 12; GLY: n = 13) *p < 0.05 in a Student’s t-test (unpaired, two-tailed).

**FIGURE 3:**
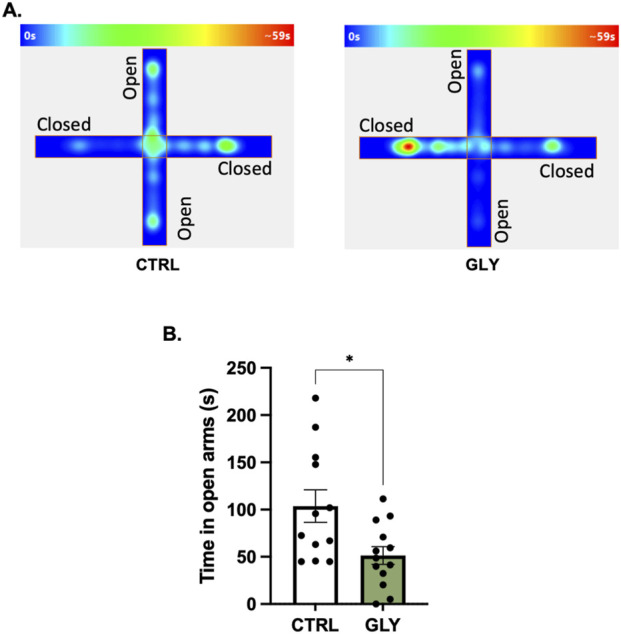
Glyphosate after 10 weeks increases anxiety-like behaviors in an elevated plus maze. **(A)** Average heat maps showing position of the animals. **(B)** Glyphosate decreases the time spent in the open arms. (CTRL: n = 12; GLY: n = 13) *p < 0.05 in a Student’s t-test (unpaired, two-tailed).

We assessed behaviors at different time points to determine the time-dependent effects of glyphosate on anxiety. Anxiety-like behaviors were assessed in two paradigms. The open field test (1 m × 1 m x 35 cm) was used after 4 weeks of glyphosate exposure (Figures 1, 2). A light was placed on the ceiling to illuminate the center more than the periphery. Lighting parameters were consistent throughout the study ensuring that control rats and experimental rats were handled and treated identically. Rats were placed in the center and left to freely behave for 5 minutes. The elevated plus maze (EPM; (La-Vu et al., 2020)) was used after 10 weeks of exposure (Figures 1, 3). The EPM consists of two sets of arms arranged perpendicular to each other raised 79 cm from the floor. Two of the arms are open (50 × 10 cm × 3.8 cm; transparent, short walls) permitting visual input of the height and proprioception to activate aversion to the open spaces (Martínez et al., 2002), and two closed arms (50 × 10 × 48 cm; opaque tall walls), with a central neutral area (10 × 10 cm) connecting the arms. Rats were placed in the center facing an open arm and left to freely behave for 5 minutes.

**FIGURE 4:**
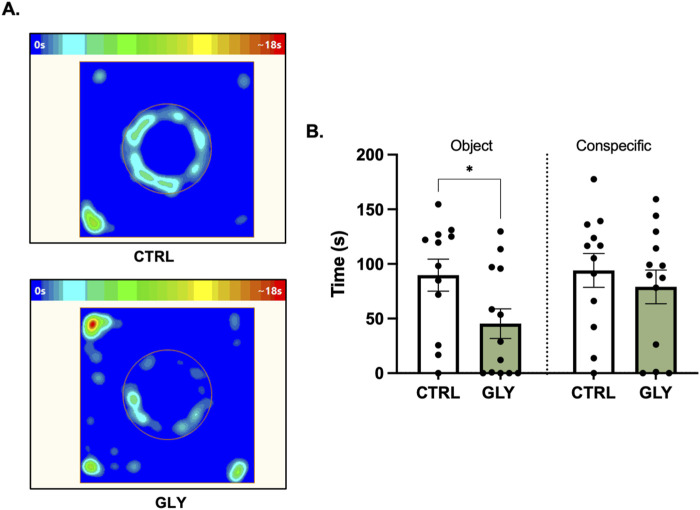
Effects of glyphosate on exploration of a novel object and conspecific after 14 weeks. **(A)** Average heat maps showing position of the animals during exploration of a novel object. **(B)** Glyphosate decreases the time interacting with a novel object but not a conspecific. (CTRL: n = 12; GLY: n = 13) *p < 0.05 in a Student’s t-test (unpaired, two-tailed).

The Social Exploration Test was used after 14 weeks of exposure (Figures 1, 4) (Bravo-Rivera et al., 2021). Rats were acclimated to the open field for 5 minutes. Next, a novel neutral object (rubber toy) was placed in the center of the open field within a mesh cage (25 cm in diameter and 28 cm tall). Rats were placed in the field and behaved freely for 3 minutes. The area surrounding the mesh cage (diameter: 12.5 cm) was defined as the exploration zone, which occupied 1,473 cm^2^ (15% of the area). The novel neutral object was then replaced with an unfamiliar naïve rat of the same age and sex as a conspecific. A total of four naïve rats were used as conspecifics. Each naïve rat was used for a maximum of seven sessions. The naïve rats were not used in any other experiment in the study. Time spent in the exploration zone was measured across 3 minutes.

### Fear conditioning and extinction

**FIGURE 5:**
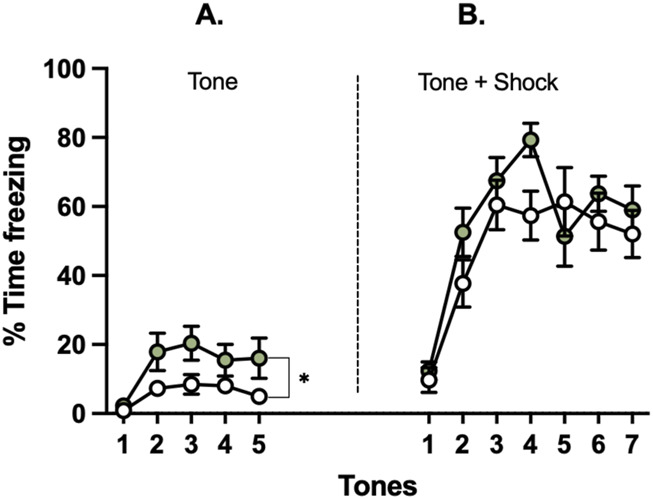
Effects of glyphosate on freezing to a novel vs. conditioned tone after 16 weeks. Glyphosate increases threat response (freezing) to **(A)** a novel neutral tone, **(B)** but not to a conditioned tone. (CTRL: n = 12; GLY: n = 13) *p < 0.05 in a 2-way ANOVA (group × time interaction).

**FIGURE 6:**
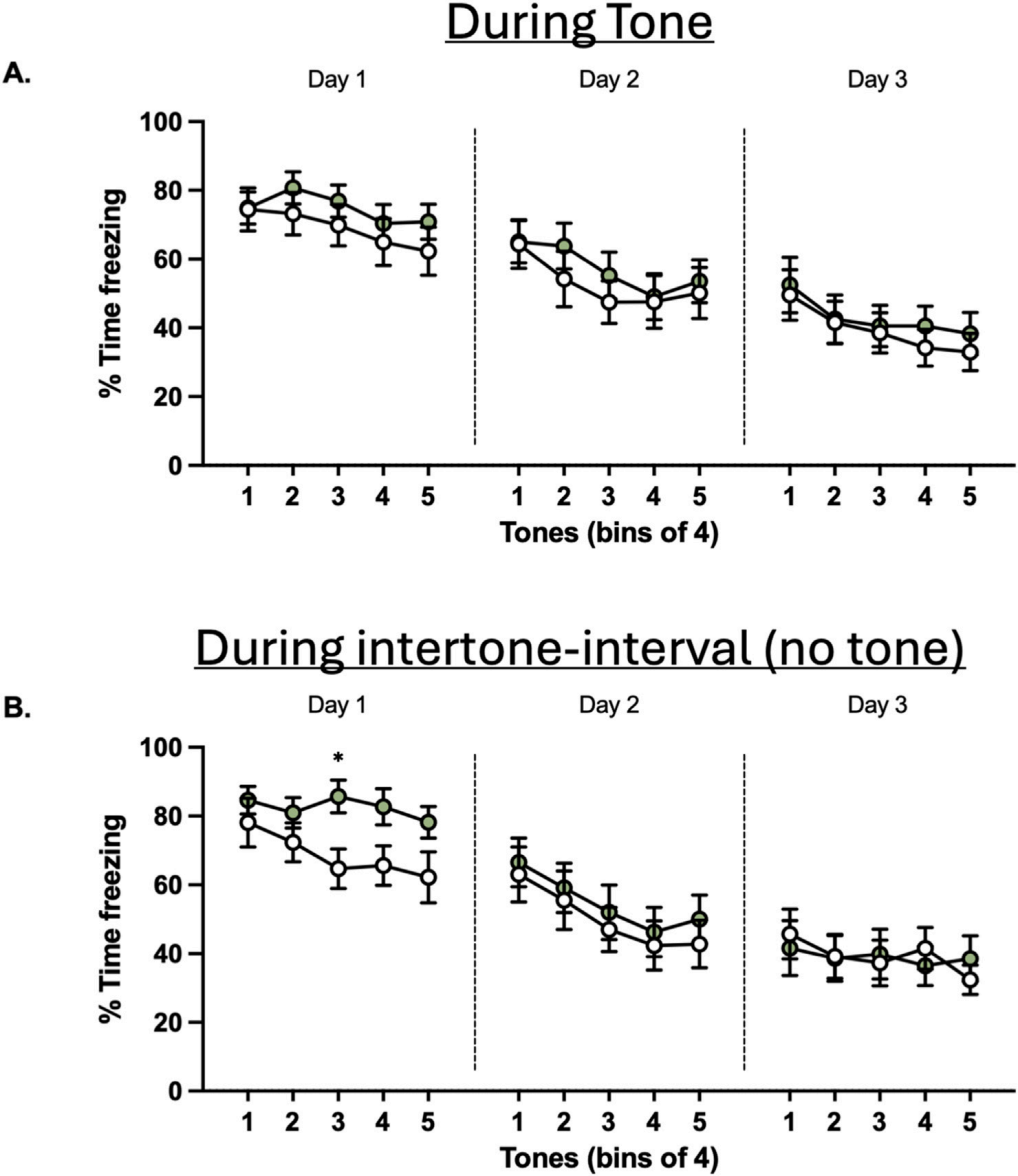
Effects of glyphosate on extinction to a conditioned tone after 16 weeks. **(A)** Glyphosate does not affect extinction of a conditioned threat response. **(B)** Glyphosate decreases extinction in the post-tone period. (CTRL: n = 12; GLY: n = 13) * p < 0.05 in a 2-way ANOVA (group x time interaction) with Bonferroni’s multiple comparisons test.

After 16 weeks of exposure (Figures 1, 5), conditioning occurred in the chamber where rats learned to press a lever. Rats were presented with five repetitions of a novel neutral tone (4 kHz, 75 dB, 30 s, 3 min ITI) followed by seven repetitions of the same tone that co-terminated with a mild foot shock (0.6 mA, 0.5 s). Freezing is defined as a lack of movement save for breathing (Blanchard and Blanchard, 1969). For extinction, 20 repetitions of the tone per day without shock were given for 3 days. Freezing was measured during the tone (Figure 6A) and the first 60-s of each post tone interval (Figure 6B). Conditioning data were analyzed as single tones, whereas extinction data were analyzed as average blocks of four tones. The apparatus was cleaned with paper towel embedded with 25% alcohol after each session.

### Immunohistochemistry and c-Fos quantification

**FIGURE 7:**
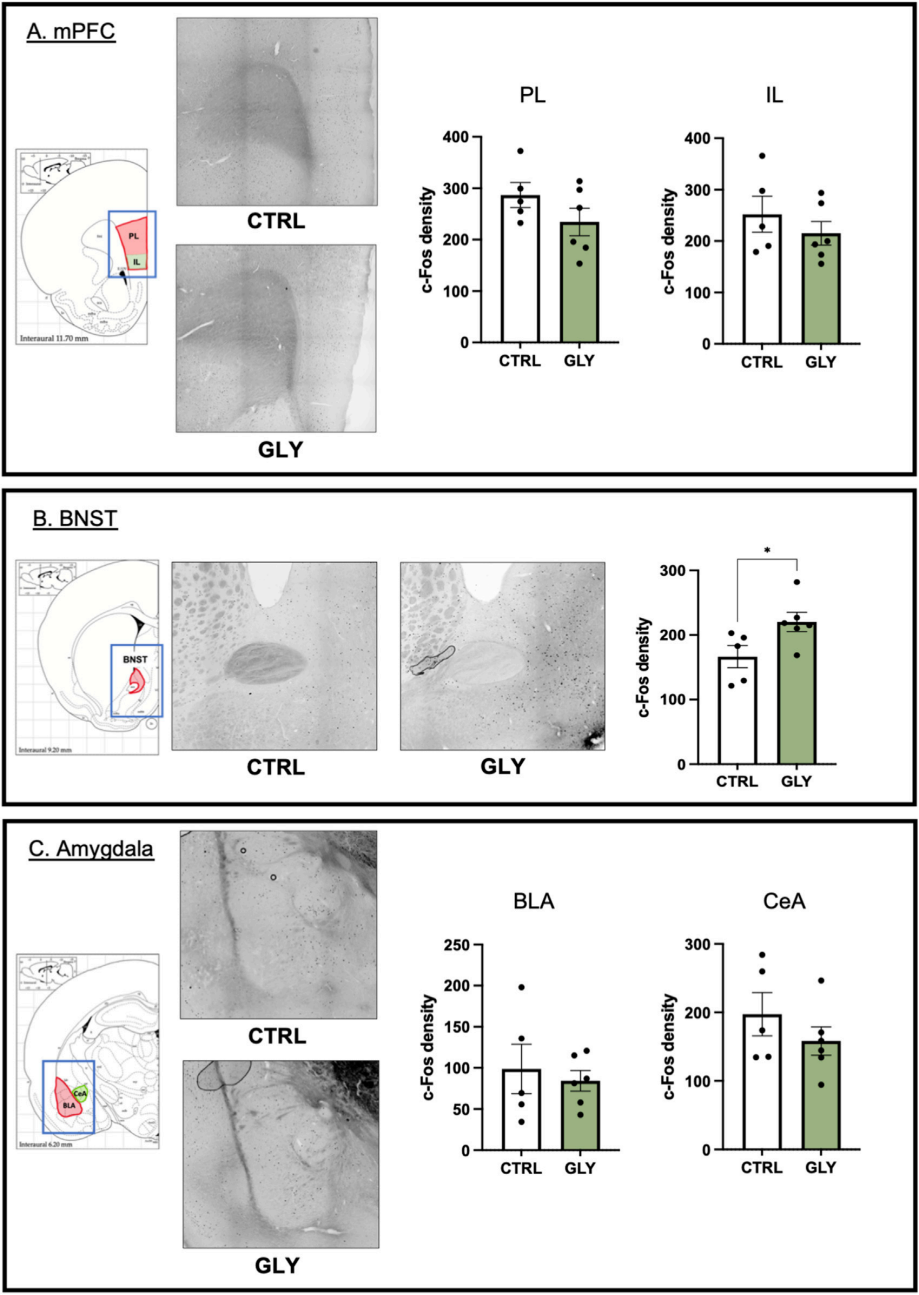
Effects of glyphosate on cellular activity. **(A)** Glyphosate does not affect c-Fos density in PL or IL of mPFC. **(B)** Glyphosate increases c-Fos density in BNST. **(C)** Glyphosate does not affect c-Fos density in BLA or CeA of the amygdala. (CTRL: n = 5; GLY: n = 6) * p < 0.05 in a Student’s t-test (unpaired, two-tailed). X10 magnification.

One-hour after the last auditory tone in extinction, rats were anesthetized with sodium pentobarbital (i.p., 450 mg/kg) and euthanized according to the American Veterinary Medical Association (AVMA) guidelines (Leary et al., 2020). Rats were transcardially perfused with 0.9% saline followed by buffered formalin. Brains were extracted, fixed in formalin and transferred to 30% sucrose in 0.1 M PBS for cryoprotection. Coronal sections (40 µm) of regions of interest [Figure 7; mPFC, (AP: +3.00 to +3.70); BNST, (AP: 0.00 to −0.50); and BLA, (AP: 3.00 to −2.00)] were cut using a cryostat (Leica, CM 1850).

Only brains with good representative sections of all regions of interest were used. Brains that displayed damage to any brain region of interest during extraction, or that were incompletely perfused, were not included in the analysis. A total of 11 brains were included (CTRL: n = 5, GLY: n = 6). Anti-c-Fos serum raised in rabbit (Cell Signaling, MA, United States; 1:1,000) was the primary antibody. Next, brain sections were washed to clear any unbound antibody. Sections were incubated with a secondary goat anti-rabbit antibody (Vector Labs; 1:500 for 1 h), followed by treatment with a Vectastain kit (Vector) for 30 min, and developed with a 3,3′-diaminobenzidine (DAB; Vector). Water stopped the development of the DAB reaction. Sections were mounted on a slide and cover-slipped (Invitrogen, MA, United States). Automatic counting for c-Fos positive cells was performed from images taken of at least one hemisphere for at least two slices (NIS Elements, Nikon, NY, United States). Total cell count was divided by the area of the region of interest to obtain a density of positive cells.

### DNA extraction from fecal boli and 16S rRNA sequence analyses

Fecal pellets were collected, and 200 mg were used for gDNA extraction using the DNeasy PowerSoil Pro Kit (QIAGEN, Germantown, MD, United States) according to instructions of the manufacturer. The 16S rRNA gene was amplified with universal 16S V3-V4 as per the earth microbiome project protocol (https://earthmicrobiome.org/protocols-and-standards/16s/) and further sequenced using Illumina MiSeq. Raw fastq files were uploaded to Qiita project ID 14486 (González et al., 2018). Quality control of amplicon sequences was performed in QIITA and to identify unclassified mitochondria and chloroplasts sequenced compared against the SILVA database (Quast et al., 2013). Taxonomy assignments were done with Greengenes two classifier (McDonald et al., 2024), and the Amplicon Sequence Variant (ASVs) from DEBLUR table were downloaded for downstream analyses. We only analyzed the microbiota at 16 weeks of glyphosate exposure using a rarefaction level of 6,200 reads per sample.

### Statistical analysis of microbial diversity

Beta diversity was assessed using Principal Coordinates Analysis (PCoA) with Bray-Curtis dissimilarity, and statistical significance was evaluated using Permutational Multivariate Analysis of Variance (PERMANOVA) and Analysis of Similarities (ANOSIM) (Anderson, 2001) in Qiime2 (Bolyen et al., 2019). Alpha diversity was calculated for Chao1 (Kuczynski et al., 2012) and Shannon (Caporaso et al., 2010) indices, with statistical differences across multiple groups assessed using the Kruskal–Wallis test in Qiime2. Plots for alpha and beta diversity were generated in RStudio (R Core Team, 2025) to visualize the results. To identify potential biomarkers, microbial features were ranked based on their ability to distinguish between the control group and the glyphosate-exposed group at 16 weeks using Random Forest analysis (Breiman, 2001). This was conducted through Microbiome Analyst (Dhariwal et al., 2017), which employs Mean Decrease Accuracy as the ranking metric. Additionally, the Firmicutes/Bacteroidetes (F/B) ratio was calculated using relative abundances of these two phyla across sample groups and visualized using bar plots in RStudio to characterize the microbiome composition.

### Data collection and analysis

Behavioral sessions were recorded with digital cameras. The open field test was used to measure distance traveled as an index of locomotor activity, and the percent of time spent in the center of the field as an index of anxiety. The elevated plus maze (EPM) was used to measure distance traveled, and time in the open arms as an index of anxiety. Distance traveled, time spent in areas of interest, and freezing were quantified from video recordings using the commercially available software ANY-maze (Stoelting, Wood Dale, IL). The normal distributions of primary behavioral data were verified using the Shapiro-Wilk Test. Data collected from the open field test and EPM were analyzed with a Student’s t-test. The time spent freezing was expressed as a percentage. Conditioning data were analyzed as single tones, whereas extinction data were analyzed as average blocks of four tones. Freezing data were analyzed with repeated-measures analysis of variance (ANOVA), followed by Tukey’s *post hoc* comparisons (GraphPad, La Jolla, CA). Gut microbiome data were analyzed with non-parametric tests Analysis of similarities (ANOSISM) and Kruskal–Wallis test.

## Results

### Glyphosate does not affect bodyweight, food consumption or water intake

Bodyweight, food consumption and water intake were measured weekly. There was no difference in bodyweight throughout the exposure period (CTRL: 492.3 g, GLY: 485.6 g; t_23_ = 0.5956, p = 0.5956; Supplementary Figure S1A). Similarly, there was no difference in food consumption (CTRL: 21.00 g, GLY: 20.38 g; t_23_ = 0.9378, p = 0.358; Supplementary Figure S1B), or water intake (CTRL: 23.7 mL, GLY: 21.4 mL; t_23_ = 1.466, p = 0.1562; Supplementary Figure S1C). Ergo, glyphosate does not affect bodyweight, and any potential changes were not masked by altered food consumption or water intake.

### Glyphosate does not affect anxiety-like behaviors after 4 weeks but increases them after 10 weeks

Rats were placed in an OFT to assess the effect of glyphosate on anxiety-like behaviors after 4 weeks (Figure 2). A Student’s t-tests showed that glyphosate did not affect the time spent in the center (CTRL: 49.01 s, GLY: 40.42 s; t_23_ = 1.155, p = 0.2601). Next, after a total of 10 weeks, rats were placed in an Elevated Plus Maze (EPM) to further test anxiety (Figure 3). A Student’s t-test showed that glyphosate decreased the time spent in the open arms (CTRL: 103.7 s, GLY: 51.35 s; t_23_ = 2.731, p = 0.0119; Figure 3B). Glyphosate did not influence locomotion (Supplementary Figure S2).

### Glyphosate decreases exploration of a novel neutral object but not a social rat after 14 weeks

Rats underwent a Social Exploration Test after 14 weeks (Figure 4). A Student’s t-test showed that glyphosate decreased the time spent exploring the novel neutral object (CTRL: 89.69 s, GLY: 45.35 s; t_23_ = 2.221, p = 0.0365). Interestingly, glyphosate did not influence the time with a social rat (CTRL: 94.03 s, GLY: 79.02 s; t_23_ = 0.6880, p > 0.05, Figure 4B). Next, we assessed for immobility and time spent in the periphery, given that decreased exploration could be due to decreased desire to explore or increased anxiety-like behaviors. Interestingly, glyphosate increased the amount of time spent immobile (CTRL: 45.66 s, GLY: 98.83 s; t_23_ = 2.468, p = 0.0215, Supplementary Figure S3A), and increased the time spent in the periphery (CTRL: 64.85 s, GLY: 118.0 s; t_23_ = 2.121, p = 0.0449, Supplementary Figure S3B).

### Glyphosate increases fear to a novel neutral tone but not a conditioned tone after 16 weeks

Animals underwent fear conditioning and extinction after 16 weeks (Figure 5). First, animals were presented with five repetitions of a novel neutral tone. A two-way ANOVA showed that glyphosate increased time freezing to presentation of the novel neutral tones prior to fear conditioning (F_(1,23)_ = 4.385; p = 0.0475). Next, the neutral tone was paired with a mild foot shock for fear conditioning (Figure 5). No difference was seen between groups (F_(1,23)_ = 1.361; p = 0.2553) or interaction of factors group x time (F_(6,138)_ = 1.608; p = 0.1493).

Finally, animals underwent fear extinction training (Figure 6). Two-way ANOVA showed no difference in freezing to the tone between groups or interaction of factors group x time on neither Days 1–3 (p > 0.05; Figure 6A). To gain further insight into anxiety, we measured time freezing in the 60-s post-tone interval during extinction (Figure 6B). A Two-way ANOVA revealed a significant difference in interaction of factors group x time (F_(4,92)_ = 2.540.; p = 0.0451) during the first day of extinction. Bonferroni’s-corrected *post hoc* test revealed that freezing was significantly increased at tone block 3 (adjusted p = 0.0417). Conversely, this difference was not seen during days 2 and 3 of extinction.

### Glyphosate increases cellular activity in BNST, but does not affect cellular activity in PL, IL, BLA or CeA

To assess the effect of glyphosate on the brain circuitry of anxiety and fear, we performed c-Fos immunohistochemistry, to measure cellular activity, on brain slices containing the prelimbic (PL) and infralimbic (IL) cortices of the medial prefrontal cortex, the bed nucleus of the stria terminalis (BNST), and the basolateral (BLA) and central (CeA) amygdala. Glyphosate did not affect cellular activity in PL (CTRL: 286.9 counts/cm^2^, GLY: 234.5 counts/cm^2^; t_9_ = 1.427, p = 0.1874) or IL (CTRL: 252.3 counts/cm^2^, GLY: 215.1 counts/cm^2^; t_9_ = 0.9192, p = 0.3819) as seen in Figure 7A, respectively. On the other hand, glyphosate increased cellular activity in BNST (CTRL: 166.5 counts/cm^2^, GLY: 220.2 counts/cm^2^; t_9_ = 2.380, p = 0.0412, Student’s T-Test) as seen in Figure 7B. Glyphosate did not affect cellular activity in BLA, (Figure 7C. CTRL: 98.66 counts/cm^2^, GLY: 84.21 counts/cm^2^; t_9_ = 0.4751, p = 0.6460), or CeA (Figure 7C. CTRL: 197.4 counts/cm^2^, GLY: 158.1 counts/cm^2^; t_9_ = 1.079, p = 0.3087).

### Glyphosate causes microbiota taxonomic changes with a decrease of *lactobacillus* genus after 16 weeks

**FIGURE 8:**
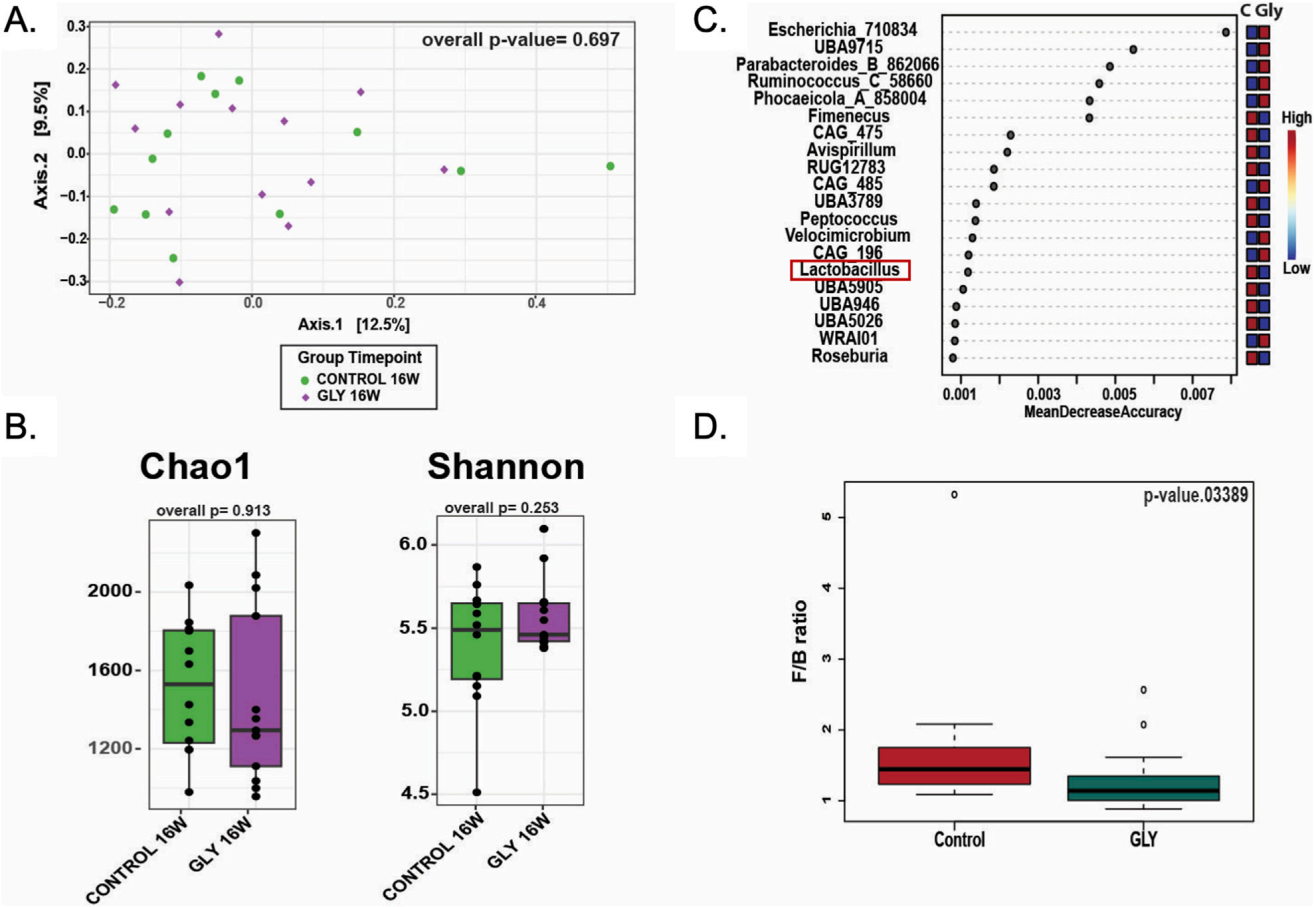
Effects of glyphosate exposure for 16 weeks on gut microbiota from fecal pellet. **(A)** Beta diversity plots showing that Glyphosate does not impact the overall composition of the gut microbiota. **(B)** Chao1 richness and Shannon index for alpha diversity did not differ between groups. **(C)** Specific taxonomic changes in the microbiota of animals treated with glyphosate included lower *Lactobacillus* abundance and increased *Escherichia*. **(D)** Firmicutes/Bacteroidetes (FB) ratio differed significantly between groups (Kruskal–Wallis p = 0.034) indicating glyphosate-induced gut dysbiosis.

After completion of all behavioral experiments, 25 fecal samples (12 control and 13 glyphosate-exposed rats at 16 weeks) were analyzed to gain insight into potential changes of gut microbiota. An average of 9,586 ± 675 sequencing reads and 1,324 ± 81 amplicon sequence variants (ASVs) per sample were yielded. No significant differences in beta diversity were observed between control and glyphosate-treated groups at week 16 (ANOSIM p > 0.05, PERMANOVA p > 0.05; Supplementary Tables S1–S3; Figure 8A). Also, alpha diversity indices, including Chao1 richness (p = 0.913) and Shannon diversity (p = 0.253), showed no significant variation between treatments (Supplementary Table; Figure 8B). Despite not showing clear diversity differences, we found specific taxonomic changes in the microbiota of rats treated with glyphosate, which included lower *Lactobacillus* abundance compared to controls (Figure 8C). In addition, the Firmicutes-to-Bacteroidetes (F/B) ratio differed significantly between control and glyphosate groups (Kruskal–Wallis p = 0.034; Figure 8D), with a reduction in Bacteroidetes with a concomitant dysbiosis associated to the glyphosate treatment.

## Discussion

We exposed rats to a dose of glyphosate considered safe by the Environmental Protection Agency (EPA, 2017). Glyphosate increased anxiety-like behavior and enhanced threat interpretation of novel neutral stimuli. Consistent with changes in behavior, we observed increased cellular activity in the anxiety-promoting region, BNST. Lastly, we demonstrate that glyphosate induces a reduction in *Lactobacillus* of the gut microbiota. In sum, our results support the idea that glyphosate should be reevaluated for safety (Vandenberg et al., 2017), and considerations could be given to evaluate its capacity to affect mental health.

### General toxicity of dose used in rats

Rat metabolism is about 6–10 times higher than that of humans (Agoston, 2017). Considering allometric scaling, a dose of 2.0 mg/kg/day in the rat corresponds to 0.2–0.3 mg/kg/day in the human, which is considered safe since it is lower than the maximum level permitted by the EPA. Of note, glyphosate had a significant effect on anxiety-like and fear-related behaviors at this low dose.

### Glyphosate induces anxiety-like behaviors in a time-dependent manner

Glyphosate increased anxiety-like behaviors as exposure period increased. We did not see increased anxiety-like behaviors in the OFT after 4 weeks, contradictory to previous reports (Ait Bali et al., 2017; Ait Bali et al., 2018; Ait Bali et al., 2022; Baier et al., 2017). This may be due to a difference in doses, since after six more weeks of exposure, glyphosate decreased time spent in the open arms of an EPM. Like the OFT, the EPM is a classic assay for anxiety (La-Vu et al., 2020). Accordingly, a minimum exposure period is likely necessary for low doses of glyphosate to accumulate in the body to increase anxiety. This could be tested in separate cohorts of rats at different time points.

Glyphosate is excreted via the kidneys and is nephrotoxic at low doses (Mesnage et al., 2015). Hence, prolonged exposure may lead to decreased excretion and accumulation of glyphosate. Accumulation could lead to detrimental effects on the central nervous system. Interestingly, studies in rodents have shown that glyphosate crosses the blood-brain barrier (Martínez and Al-Ahmad, 2019; Winstone et al., 2022). A logical next step would be to evaluate if exposure to glyphosate at a dose of 2.0 mg/kg leads to detection of glyphosate and metabolites in the brain.

### Glyphosate increases threat response to neutral, but not emotionally-relevant stimuli

Increased anxiety creates a conflict between the drive to explore and avoid potential threats (Ennaceur and Chazot, 2016). Considering this, we assessed the animals’ response to novel stimuli in two ways. First, animals were presented with a neutral inanimate object. Here, animals exposed to glyphosate displayed reduced time interacting (Figure 4), as well as increased immobility and thigmotaxis (Supplementary Figure S3). On the other hand, when the animals interacted with a conspecific (positive valence), no difference was seen in time spent exploring (Figure 4). Second, when presented with a neutral auditory stimulus, glyphosate increased freezing (Figure 5A). In contrast, when the tone was associated to a foot-shock (negative valence), there was no difference in freezing (Figure 5B). These results imply that glyphosate causes the animals to process neutral stimuli as possible threats, but does not affect their response to emotionally-relevant stimuli. This is translationally-relevant since an increase in avoidance behaviors is characteristic of trauma-related and anxiety disorders (DSM-V, 2013).

### Glyphosate induces a sustained threat response, which is overcome with continued extinction

We did not observe differences in freezing during the tone throughout extinction. One possibility is that extinction to the conditioned tone recruits brain regions that are not sensitive to glyphosate. Consistent with this, we saw no difference in cellular activity in neither subregions of the medial prefrontal cortex nor the amygdala. Given the importance of fear responses to survival, it is expected that the fear circuitry would show significant resilience to disruption in cellular activity, particularly at the low dose of glyphosate used in this study.

Glyphosate impairs the cognitive flexibility required to identify absence of a learned threat, as observed by a sustained threat response following the termination of each presentation to the auditory stimulus (post-tone, Figure 6B). This is characteristic of trauma- and stress-related disorders, raising the question of the effect of environmental contaminants on the response to traumatic events. This impairment was overcome with continued extinction.

### Glyphosate alters neural activity in the neurocircuitry of anxiety

We observed increased cellular activity in the bed nucleus of the stria terminalis (BNST) which is implicated in the promotion of anxiety (Goode et al., 2020). Given the role of BNST in threat interpretation, we suggest that glyphosate increases anxiety in the elevated plus maze and threat response to novel, neutral stimuli by altering cellular activity in anxiety promoting regions. Furthermore, we suggest that the increased freezing during the post-tone period represents a state of hypervigilance sustained by the BNST, since BNST has been shown to play a minor role in fear learning to contextual, but not auditory, cues (Goode et al., 2019; 2020). A consideration about the present study is that the timing of increased cellular activity in BNST most closely corresponded to the behavioral time point at the end of fear extinction (Figure 1). Future work could assess the effect of glyphosate over time on activity in BNST in separate cohorts of animals.

### Glyphosate induces gut dysbiosis with a reduction in *Lactobacillus*

Glyphosate targets the shikimate pathway, which is absent in mammals but present in bacteria (Williams et al., 2000; Mir et al., 2013). Previous studies have shown that glyphosate alters gut microbial composition as well as the *Firmicutes/Bacteroidetes* (F/B) ratio, an indicator of gut dysbiosis (Ait Bali et al., 2018; Mao et al., 2018; Dechartres et al., 2019; Tang et al., 2020b). Similarly, in the present study, glyphosate at 2.0 mg/kg daily altered the F/B ratio despite differences in dosage, formulations, and age (Tamburini et al., 2016; Yassour et al., 2016; Lozano et al., 2018; Wang et al., 2020).

Gut microbiota are necessary to produce the essential amino acid tryptophan (Paley, 2019). Tryptophan is the precursor in the production of the neurotransmitter serotonin, which is critical for modulating anxiety and fear behaviors (Ravenelle et al., 2025). The *Lactobacillus* genus has been implicated in the production of serotonin from tryptophan (Gao et al., 2020; Yong et al., 2020), and alterations in its abundance influence anxiety (Bravo et al., 2011; Cowan et al., 2019; Barros-Santos et al., 2020; Tette et al., 2022). In the present study, glyphosate decreased *Lactobacillus* abundance, a marker of eubiosis in the gut, like previous work (Ait Bali et al., 2018; Lozano et al., 2018; Mao et al., 2018; Tang et al., 2020b). This highlights a susceptibility of *Lactobacillus* to glyphosate. A possible explanation for this is the chelating effect of glyphosate on manganese (Mn) (Bernards et al., 2005; Cakmak et al., 2009; Mertens et al., 2018). Species from the *Lactobacillus* require more Mn than other species (Archibald and Fridovich, 1981; Peacock and Hassan, 2021), since they utilize Mn for protection against oxidation damage. Thus, decreased availability of Mn because of glyphosate exposure may leave *Lactobacillus* vulnerable to oxidative stress. To our knowledge, only one study evaluated the effect of glyphosate on plasma Mn concentration in vertebrates. Dechartres and colleagues (2019) revealed that low dose glyphosate did not alter Mn levels in plasma. Unfortunately, Mn levels in the gut were not evaluated. Further research into the effect of glyphosate on *Lactobacillus* and manganese levels in the gut are needed.

Importantly, our work evaluated the effect of glyphosate on microbiota composition at one time point, which was after the total 16 weeks of exposure. Moreover, our data analysis represented populations of bacteria obtained from fecal pellets, but not gut tissue. This is important because the mucosal lining of the gut contains copious amounts of bacteria that are not necessarily present in the fecal pellet (Tang et al., 2020a). Consequently, it is possible that the alterations we observed do not entirely explain our behavioral findings. To address these issues, more work is needed to evaluate the progressive changes in gut microbial composition caused by glyphosate through time.

Recent metabolomic work demonstrated that glyphosate decreases levels of tryptophan in feces albeit only at higher doses (Hsiao et al., 2024). The lack of effect at lower doses reported by Hsiao and colleagues may be due to higher metabolic rates in mice *versus* rats. Moreover, previous work shows decreased serotonin content in the brain of rodents exposed to glyphosate (Ait Bali et al., 2017; Martínez et al., 2018), emphasizing the relationship between decreased *lactobacillus* with serotonin in the brain.

There is a set of literature demonstrating sex differences in behavioral outputs from fear paradigms and gut microbiome analysis. Specifically, female rats tend to display less freezing and more “darting” than male rats in fear conditioning (Colom-Lapetina et al., 2019). For this reason, combining both sexes in a fear conditioning experiment may mask any potential differences. Furthermore, evidence suggests that the response of the gut microbiome to glyphosate is different in males *versus* females (Lozano et al., 2018). For these well-established reasons of sex differences, we focused on male rats in the current set of experiments. Therefore, outcomes observed in the current study may not necessarily extrapolate to females. Indeed, future work can focus on the influence of glyphosate on behavior and microbiome in female rats.

### Putting it all together

**FIGURE 9:**
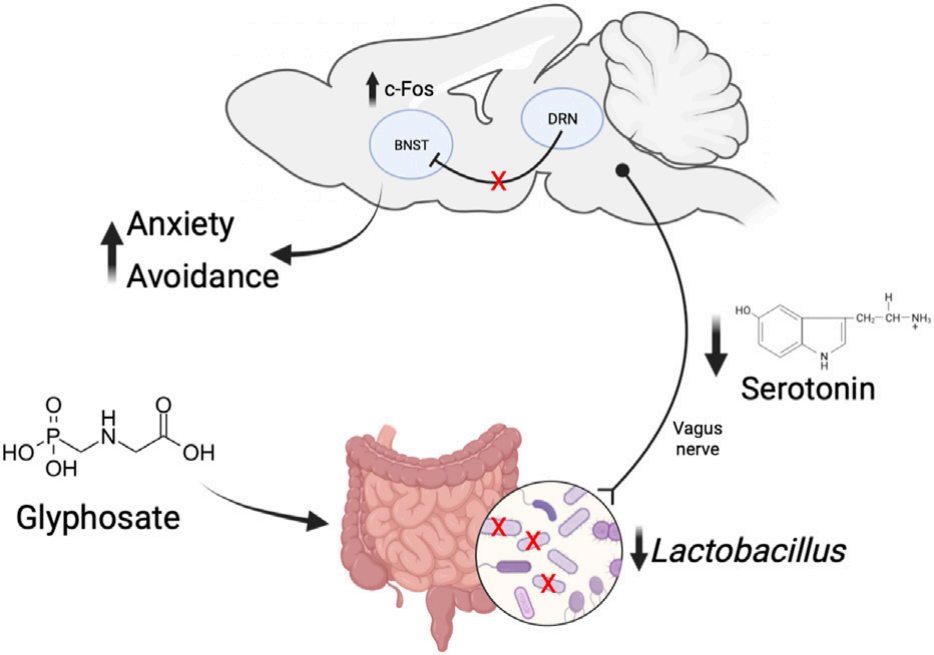
Proposed mechanism of how glyphosate increases anxiety. Glyphosate decreases the abundance of *Lactobacillus* bacteria. This results in decreased production and absorption of serotonin. Decreased serotonin in the dorsal raphe nucleus leads to a loss of modulation of cellular activity in the bed nucleus of the stria terminalis (BNST). The increased activity in BNST causes increased anxiety-like behavior and avoidance.

Figure 9 proposes a mechanism by which glyphosate increases anxiety. Previous work has shown that glyphosate decreases activity in serotonergic neurons in the dorsal raphe nucleus (Ait Bali et al., 2017). It is possible that loss of inhibitory serotonergic projections to BNST leads to the increased activity in this brain region, ultimately promoting anxiety-like behaviors and avoidance. We propose that the loss of serotonergic activity seen with glyphosate may be due to glyphosate decreasing the abundance of *Lactobacillus* bacteria, resulting in decreased production and absorption of tryptophan and subsequent serotonin.

Gut microbiota influences the brain directly. Specifically, the vagus nerve is the main afferent pathway from the gut to the brain (Schneider et al., 2024). Treatment with *Lactobacillus* probiotics alters emotional behaviors, and this effect is absent in vagotomized animals (Bravo et al., 2011; Pérez-Burgos et al., 2013; Liu et al., 2021). Hence, it is possible that the effects seen in this study are due to disruptions in *Lactobacillus* abundance and subsequent changes in brain activity via the vagus nerve.

## Data Availability

The datasets presented in this study are available in public online repositories. Specifically, the 16S rRNA sequencing data can be accessed from the European Nucleotide Archive (ENA) under the study accession number PRJEB96752. The data are available at the following link: https://www.ebi.ac.uk/ena/browser/view/PRJEB96752. Specific raw behavioral data can be distributed upon request via Google Drive.

## Supplementary material

SUPPLEMENTARY FIGURE S1 Glyphosate does not affect: **(A)** average body weight (p = 0.5956), **(B)** average daily food consumption (p = 0.358), nor **(C)** average daily water intake (p = 0.1562). (CTRL: n = 12; GLY: n = 13). All data were analyzed with Student’s t-test (unpaired, two-tailed).

SUPPLEMENTARY FIGURE S2 Effects of glyphosate on exploration of a novel object after 14 weeks. In the presence of a novel object, glyphosate increases **(A)** time immobile and **(B)** time in the periphery. (CTRL: n = 12; GLY: n = 13) *p < 0.05 in a Student’s t-test (unpaired, two-tailed).

SUPPLEMENTARY FIGURE S3 Glyphosate did not influence locomotion, as indicated by no change in distance traveled in either the **(A)**. Open Field Test, or **(B)**. Elevated Plus Maze (CTRL: n = 12; GLY: n = 13) *p > 0.05 in a Student’s t-test (unpaired, two-tailed).

SUPPLEMENTARY TABLE S1 Summary of counts, average and standard deviation for reads and ASVs across treatments, timepoint 16W, fear index and extinction index.

SUPPLEMENTARY TABLE S2 Beta diversity comparisons showed no significant differences in overall community structure between control and glyphosate-treated groups at week 16.

SUPPLEMENTARY TABLE S3 Alpha diversity metrics did not differ between the two treatment groups at week 16.
