# Supplementary figures and images for: Exposure to the herbicide glyphosate leads to inappropriate threat responses and alters gut microbial composition
Source: Front Toxicol. 2025 Nov 5;7:1704231. doi: 10.3389/ftox.2025.1704231 (PMC12626795; doi:10.3389/ftox.2025.1704231)

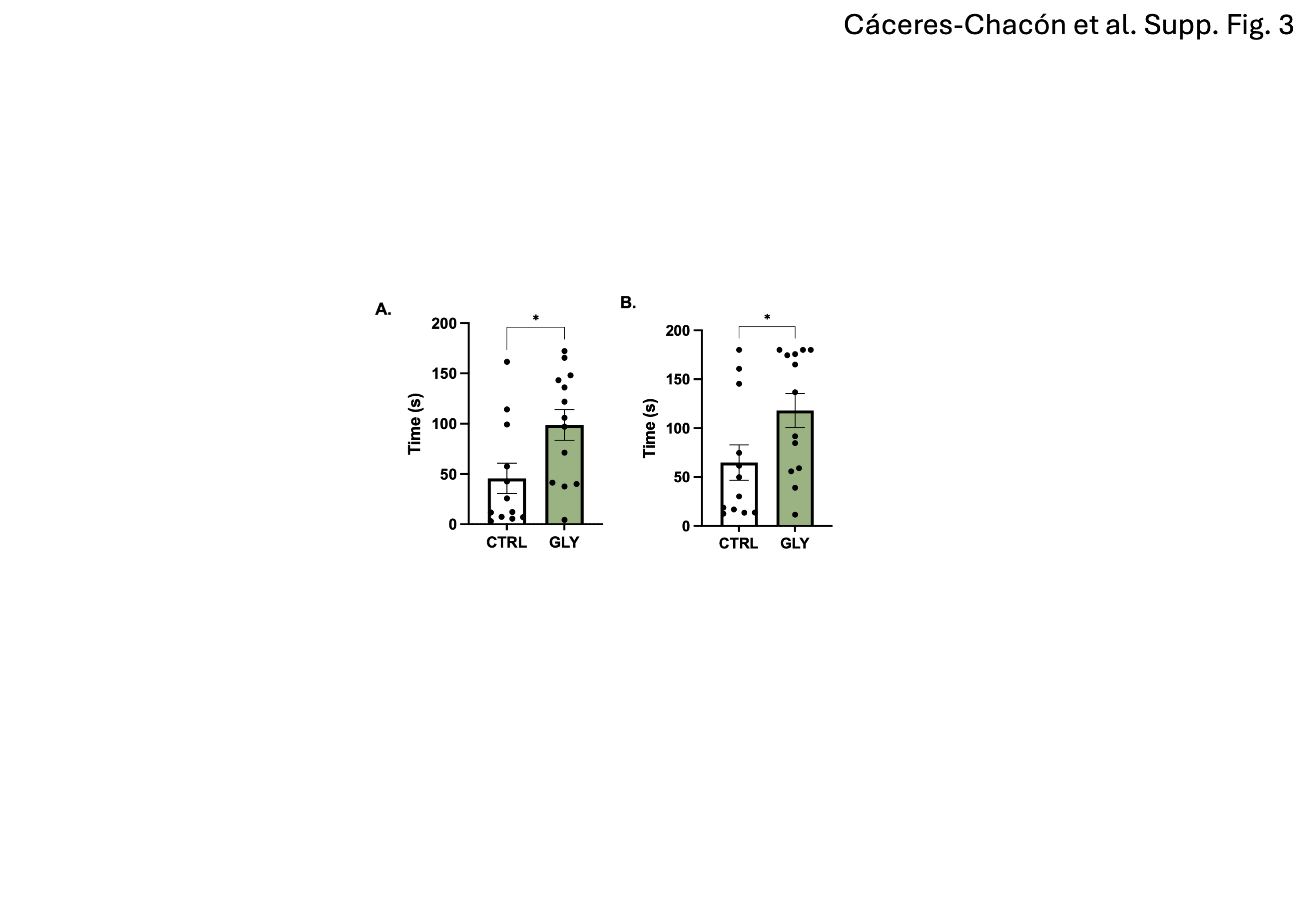

Supplement: Supplementary file 1 [file Image3.tiff]

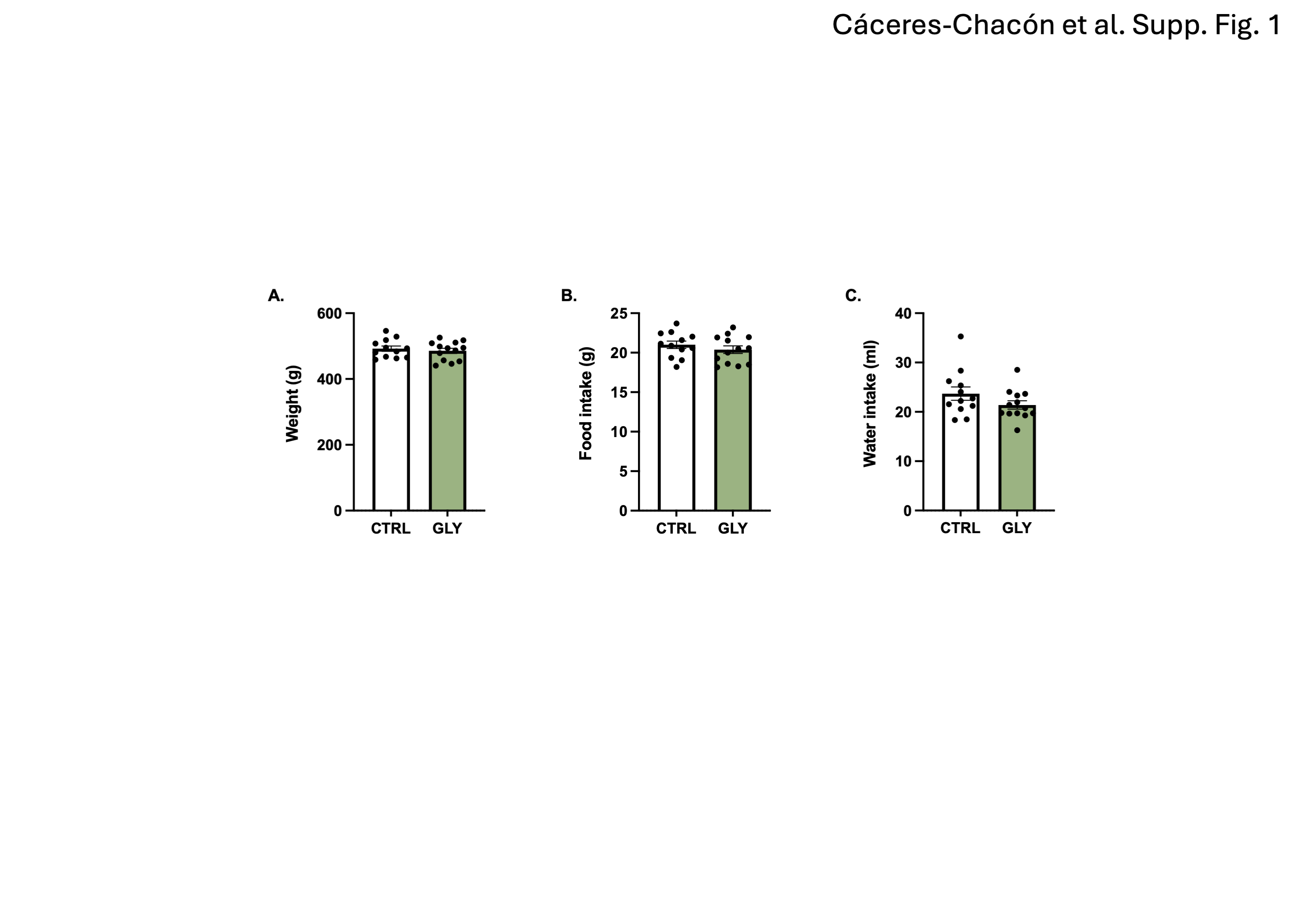

Supplement: Supplementary file 2 [file Image1.tiff]

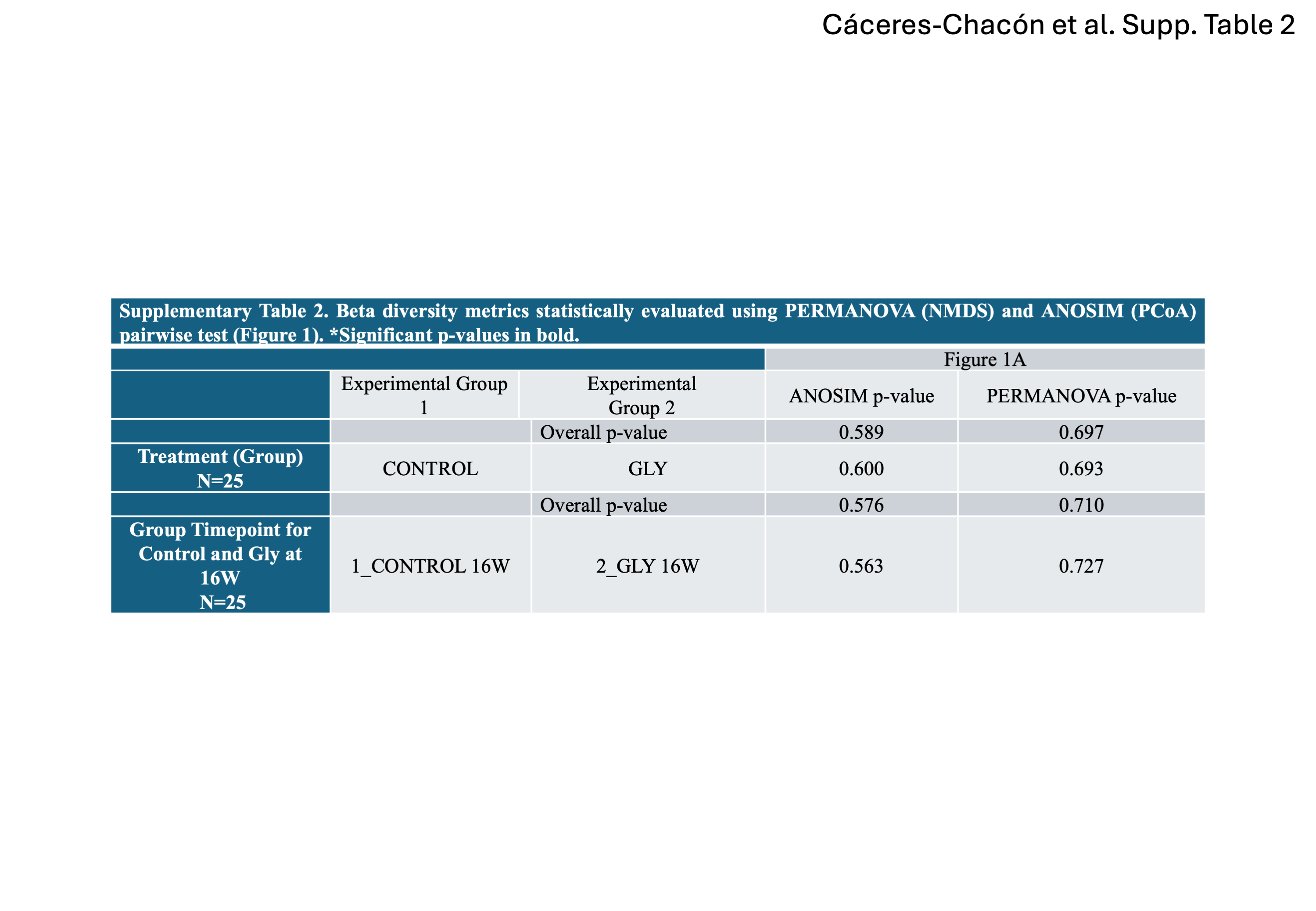

Supplement: Supplementary file 3 [file Image5.tiff]

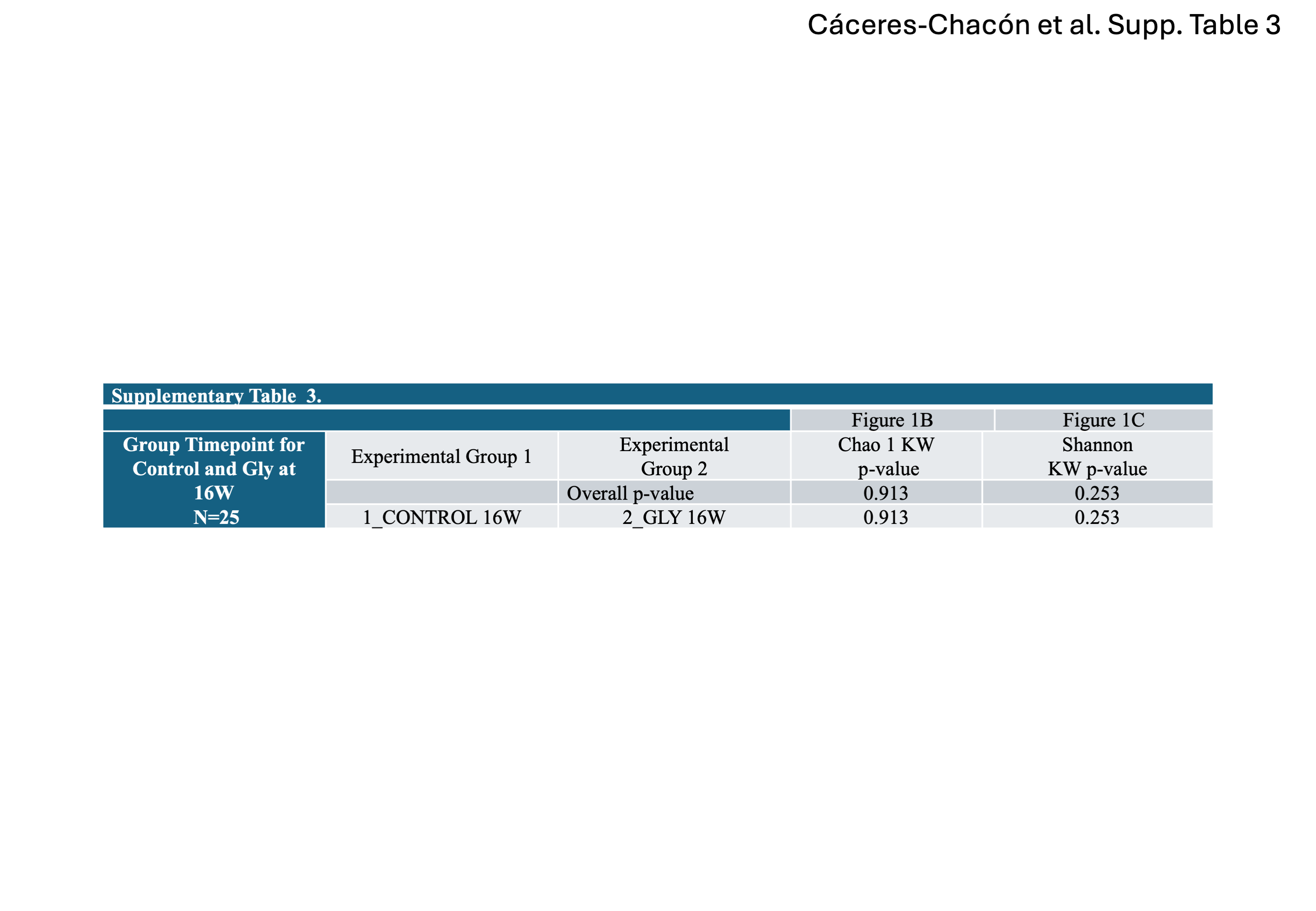

Supplement: Supplementary file 4 [file Image6.tiff]

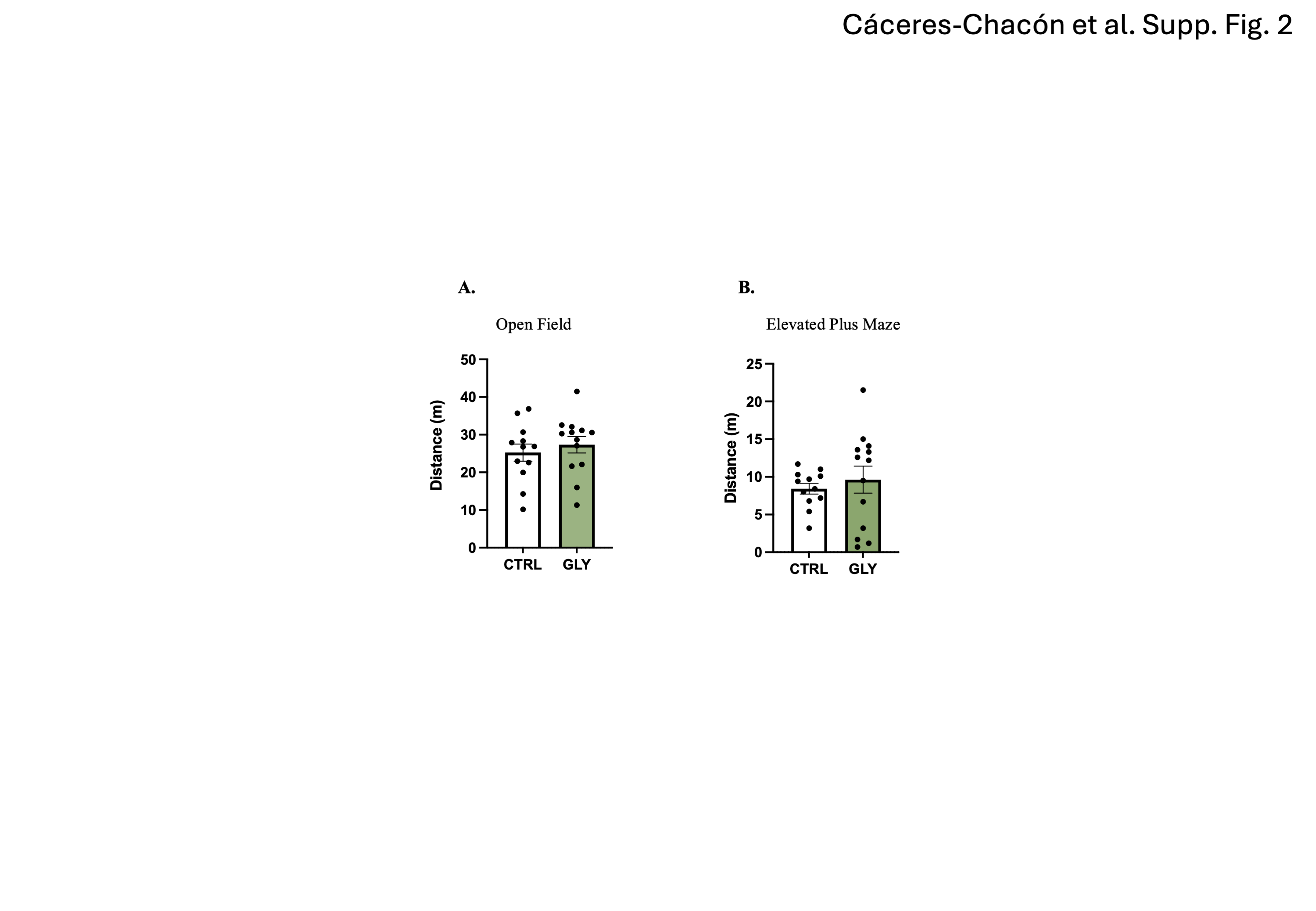

Supplement: Supplementary file 5 [file Image2.tiff]

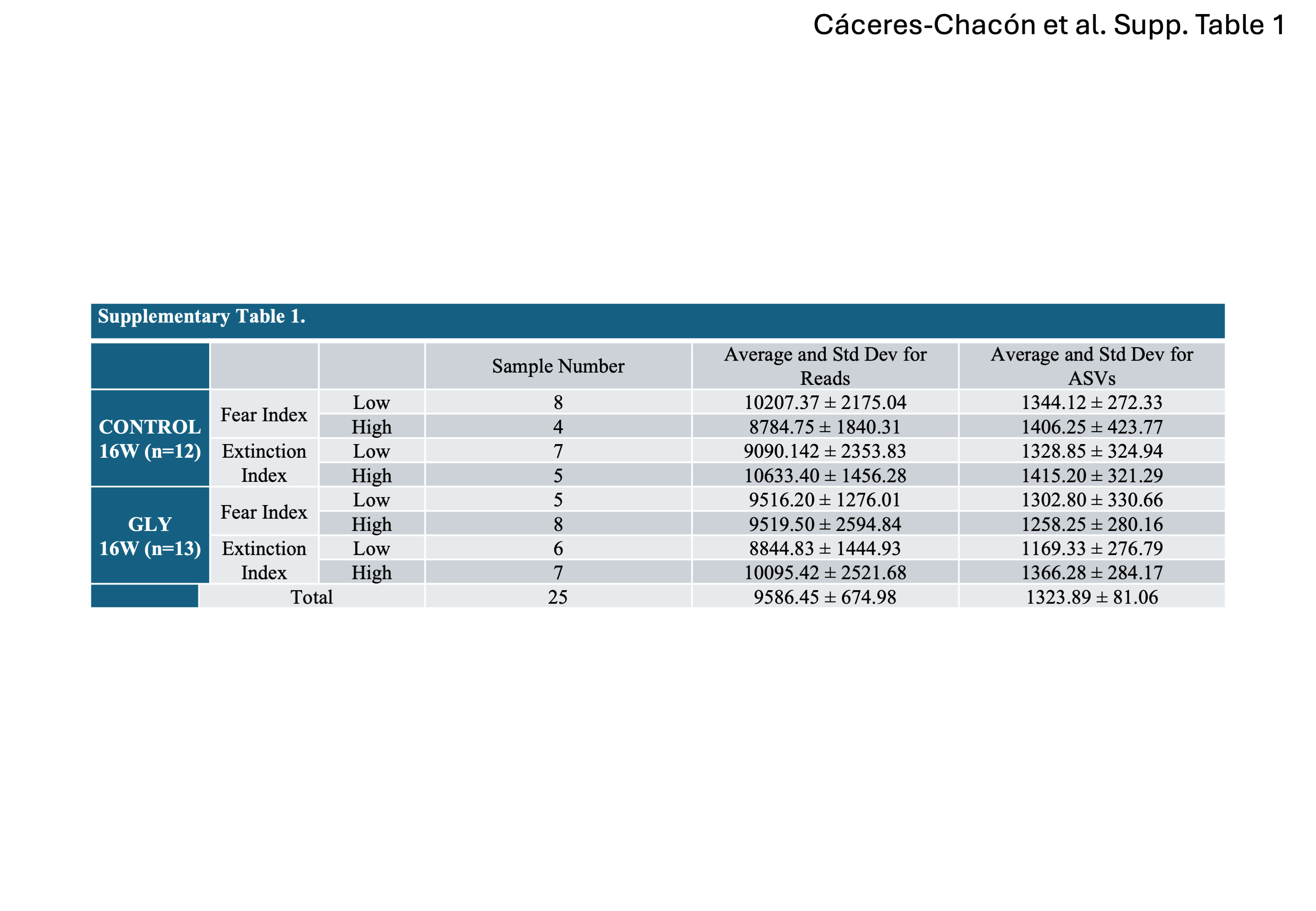

Supplement: Supplementary file 6 [file Image4.tiff]
